# Pea Protein Isolates: From Extraction to Functionality

**DOI:** 10.3390/molecules30234650

**Published:** 2025-12-03

**Authors:** Joanna Harasym, Oliwia Paroń, Ewa Pejcz

**Affiliations:** 1 Adaptive Food Systems Accelerator-Science Centre, Wroclaw University of Economics and Business, Komandorska 118/120, 53-345 Wroclaw, Poland; joanna.harasym@ue.wroc.pl; 2 Department of Biotechnology and Food Analysis, Wroclaw University of Economics and Business, Komadorska 118/120, 53-345 Wroclaw, Poland; oliwia.paron@ue.wroc.pl

**Keywords:** pea protein isolate, *Pisum sativum*, extraction, fractionation, emulsification, gelation, modifications

## Abstract

Pea protein isolates (PPIs) from *Pisum sativum* have emerged as strategic ingredients at the interface of nutrition, sustainability, and functional food design. This review synthesizes advances linking isolation procedures with molecular structure and techno-functional performance. We compare alkaline extraction–isoelectric precipitation with wet and dry fractionation, as well as green/fermentation-assisted methods, highlighting the purity–functionality trade-offs driven by denaturation, aggregation, and the removal of anti-nutritional factors. We relate globulin composition (vicilin/legumin ratio), secondary/tertiary structure, and disulfide chemistry to interfacial activity, solubility, gelation thresholds, and long-term emulsion stability. Structure-guided engineering strategies are critically evaluated, including enzymatic hydrolysis, deamidation, transglutaminase cross-linking, ultrasound, high-pressure homogenization, pH shifting, cold plasma, and selected chemical/glycation approaches. Application case studies cover high-moisture texturization for meat analogues, emulsion and Pickering systems, fermented dairy alternatives, edible films, and bioactive peptide-oriented nutraceuticals. We identify bottlenecks—weak native gel networks, off-flavors, acidic pH performance, and batch variability—and outline process controls and synergistic modifications that close functionality gaps relative to animal proteins. Finally, we discuss sustainability and biorefinery opportunities that valorize soluble peptide streams alongside globulin-rich isolates. By integrating extraction, structure, and function, the review provides a roadmap for designing PPI with predictable, application-specific performance.

## 1. Introduction

*Pisum sativum* proteins (pea protein isolates, PPIs) have recently been positioned as a critical focus in food science and ingredient technology due to the growing demand for sustainable, plant-based protein alternatives. Peas represent a rich source of high-quality protein, distinguished by their favorable amino acid composition and digestibility [1,2,3]. The protein content of peas plays a crucial role, as it is classified as a high-quality plant-based protein due to its essential amino acid profile. They contain a high concentration of lysine, an essential amino acid often deficient in cereal grains, which is why peas are frequently consumed alongside grains to ensure a complete set of essential amino acids [4,5]. The presence of leucine is particularly significant as it stimulates muscle protein synthesis and aids in recovery following physical exertion, making it valuable for athletes and individuals engaged in resistance training [2]. The protein digestibility corrected amino acid score (PDCAAS) for pea protein ranges between 0.83 and 0.91 [1,6].

Beyond its macronutritional value, pea protein offers significant advantages due to its low allergenicity, providing a safer alternative for individuals sensitive to common food allergens such as dairy, soy, or gluten [1,2]. This hypoallergenic profile enhances its appeal, especially in contexts where soy or gluten proteins are restricted, especially since research has also demonstrated its considerable nutraceutical potential.

Peptides derived from pea protein exhibit potent antioxidant capabilities, acting to scavenge free radicals and thereby reducing oxidative stress [3]. Furthermore, these peptides have been shown to inhibit angiotensin-converting enzyme (ACE) activity, which may lead to blood pressure-lowering effects and cardiovascular health benefits [3,7]. Pea protein also demonstrates a positive modulation of gut microbiota, particularly by enhancing populations of beneficial bacteria, such as Lactobacillus and Bifidobacterium [2].

The sustainability aspect is a strong point of pea cultivation; it requires comparatively less water and nitrogen fertilizer input and has positive rotational crop effects, contributing to environmental benefits while providing an economically affordable source of protein [6,7]. Historically, plant proteins, including pea protein, have played a secondary role to animal proteins in food formulation primarily due to functional limitations such as lower solubility and gelation capabilities. However, recent shifts toward sustainability and increasing production cost pressures for animal proteins have driven heightened interest in plant-based proteins, with pea protein emerging as a key player in this sector [8]. From a production standpoint, peas rank fourth globally among legumes in food production, highlighting their importance as a raw material [7].

Comparatively, pea protein offers advantages over other plant proteins, such as soy, wheat, and rice, due to its balanced amino acid composition, combined with lower allergenicity and the absence of a genetically modified organism (GMO) label [9]. While soy remains a prevalent protein source globally, concerns about allergies and GMOs have propelled research into pea protein as a preferred alternative. The global market for pea protein has grown steadily, reflecting its integration into various food products, including dairy alternatives, meat analogues, and emulsified systems, owing to technological advancements that improve its functional properties [8].

Table 1 summarizes key compositional, functional, and environmental indicators. As shown, pea protein isolate (PPI) exhibits an intermediate amino acid profile and digestibility index (PDCAAS ~0.81–0.93), positioning it between soy and cereal proteins, while maintaining low allergenicity and a significantly lower environmental footprint compared to whey. Although its globular structure limits solubility and gelation performance, recent bioprocessing innovations aim to narrow the gap with animal-derived proteins through enzymatic and physical modifications.

While the intrinsic nutritional quality of pea protein is high, achieving optimal functional performance requires addressing certain inherent limitations. The presence of anti-nutritional factors (ANFs), such as phytic acid, trypsin inhibitors, and α-galactosides, necessitates robust processing protocols [23]. The full nutritional bioavailability can only be realized by effectively mitigating or removing these ANFs during isolation. Moreover, comparative performance data suggest complexity in the application. In nutritional studies concerning muscle recovery and strength gains following high-intensity functional training, pea protein yields similar outcomes to whey protein [24].

However, when considering technological applications such as gelation or film formation, its performance often lags significantly behind animal counterparts.

The fundamental challenge is the limitation of globular proteins. Pea proteins generally exhibit significantly weaker gelling properties compared to animal proteins [25]. The intrinsic globular nature of the PPI structure renders it challenging to form robust, self-supporting viscoelastic networks under moderate conditions. This differential indicates that while pea protein is highly successful in metabolic and nutritional roles, achieving functional parity in physical applications requires extensive structural engineering and modification to address its native physicochemical limitations. The purity–functionality trade-off in isolation represents a critical decision point, where methods that maximize intact protein yield, such as harsh alkaline extraction–isoelectric precipitation (AE-IEP), may compromise functional performance through structural damage.

This review focuses on the multifaceted aspects of pea proteins, analyzing isolation techniques, molecular and structural characteristics, modification strategies, and functional capacities, including emulsification and gelation, as well as advanced applications in food systems. Central to the manuscript is the discussion of challenges such as poor solubility and off-flavor issues and strategies to overcome these challenges via structural modification techniques, including enzymatic, physical, and chemical methods. The review highlights that native pea protein structure dictates both its limitations and the engineering strategies needed to overcome them, progressing from isolation technologies through modification approaches to advanced food applications, with emphasis on structure-guided engineering approaches and biorefinery perspectives [2,6,8].

## 2. Isolation Technologies

The methods utilized for isolating pea protein impact the resultant structural integrity and, consequently, the techno-functional capabilities of the final product. Extracting high-purity protein from the dry weight of pea seeds, which contain 59.32–69.59% carbohydrates [4,5], requires methodologies that efficiently separate protein from starch, fiber, and soluble compounds.

### 2.1. Alkaline Extraction–Isoelectric Precipitation

The alkaline extraction followed by isoelectric precipitation (AE-IEP) method represents the industrial standard for commercial PPI production [26]. Traditional pea protein extraction predominantly employs this approach, which solubilizes proteins under alkaline conditions, typically utilizing pH levels between 8 and 9, to separate the protein fraction from insoluble components like starch and fiber [26,27]. Subsequent precipitation occurs by adjusting the pH to the protein’s isoelectric point (approximately pH 4.5), which effectively removes remaining soluble compounds and concentrates on the protein. Optimized AE-IEP protocols yield high protein purity, often reported in the range of 83.33–84.67% on a dry weight basis (dwb) [26,28]. The process enables effective separation from starch and fiber, yielding isolates with high protein content and reasonable functional properties.

However, the severity of the AE-IEP process introduces a significant constraint on the resulting functional profile. High alkalinity, particularly when coupled with adverse heat treatment during processing, can induce irreversible protein denaturation and polymerization [26]. High pH and heat exposure during extraction can induce partial denaturation, affecting solubility and emulsifying capacity [27]. This structural compromise negatively impacts key functionalities, such as solubility and emulsification capacity, underscoring a fundamental trade-off between purity and functionality inherent to conventional extraction. Differences in critical process variables—including the specific pH level used (e.g., pH 8.5, 9.0, or 9.5), holding time, and drying conditions—all contribute to variations in the final protein profile and functional outcome [26,28]. Spray drying conditions, for instance, affect surface hydrophobicity and particle size, which in turn impact the size and stability of emulsion droplets. Higher pH levels favor increased solubility and emulsification properties [29].

**Figure 1:**
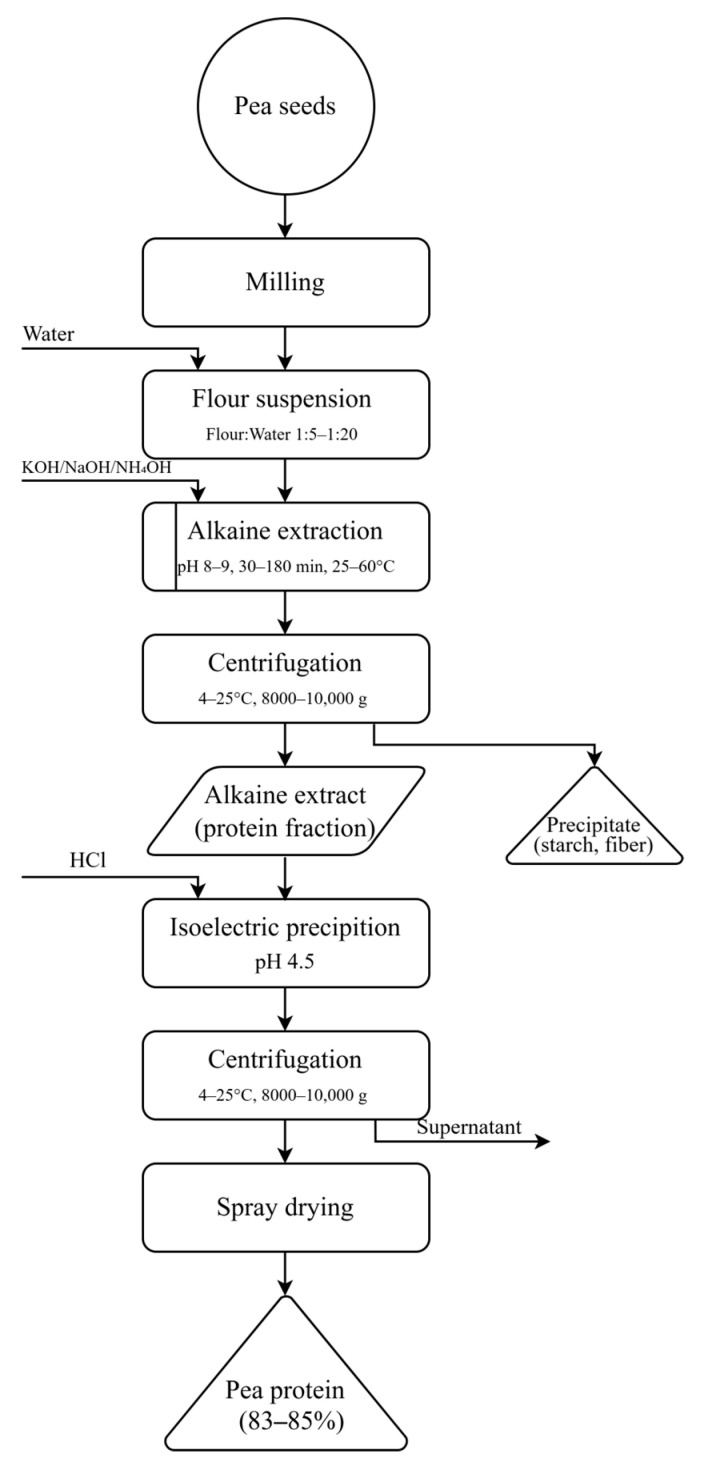
Process flow of alkaline extraction–isoelectric precipitation (AE–IEP) for pea protein isolate (PPI) preparation, showing major processing steps and conditions.

The conventional alkaline extraction–isoelectric precipitation (AE–IEP) method remains the predominant approach for producing pea protein isolates, despite its inherent limitations. Figure 1 schematically illustrates the main processing stages and critical parameters influencing protein yield and functionality, emphasizing the points at which denaturation and structural modifications are most likely to occur.

### 2.2. Wet and Dry Fractionation Methods

To circumvent the structural damage associated with AE-IEP, alternative fractionation methods are employed to produce a concentration with enhanced functional properties. Aqueous fractionation and ultrafiltration, including wet fractionation, have been shown to produce protein concentrates with purity levels of approximately 75%. Wet fractionation methods typically yield isolates with higher purity (approximately 80% protein content), but they involve significant water usage and may alter the native protein structure. These methods lead to isolates exhibiting enhanced solubility, improved emulsification, and superior foaming properties. For instance, certain aqueous/ultrafiltration processes can achieve purities of the globulin fraction exceeding 90% [28].

Dry fractionation via air classification provides a chemical-free, environmentally friendly, and cost-effective alternative, separating flour based on particle size and density without the use of solvents. The dry fractionated pea protein (DFPP) has a lower protein content (approximately 50%) but preserves native protein structures better due to milder processing conditions [30]. DFPP also retains insoluble fibers, which can confer additional functional benefits such as gelling and texture enhancement in food systems. Dry fractionated pea protein exhibits higher solubility and stronger mechanical gel properties compared to wet-extracted isolates, with gel strength nearly twofold higher at pH 7 [30]. Heat-induced gels reveal phase separation with embedded fiber-like structures contributing to mechanical reinforcement, highlighting the functional role of the insoluble polysaccharide fraction in gel network formation.

**Figure 2:**
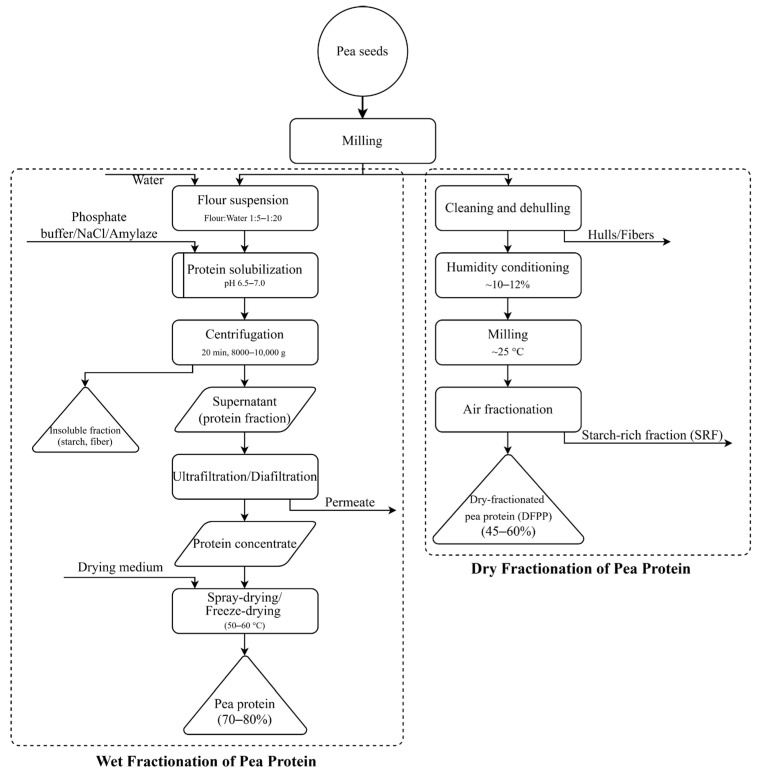
Schematic comparison of wet and dry fractionation processes used for pea protein production, showing key operational steps and typical protein yields.

In contrast, traditional salt extraction, which uses salt solubilization followed by salting out, is generally deemed industrially impractical. Salt extraction and mild fractionation techniques involve solubilizing proteins in salt solutions (e.g., NaCl) that extract salt-soluble proteins before precipitation or membrane separation. These approaches are tailored to preserve protein functionality and reduce denaturation. However, this method yields high waste streams, requires excessive water usage, and produces salty isolates that necessitate intensive washing and diafiltration steps [26]. To illustrate the broader context of pea protein production approaches, Figure 2 provides a schematic comparison of wet and dry fractionation processes, highlighting the principal operational steps, processing conditions, and typical protein yields.

### 2.3. Green and Fermentation-Assisted Extraction

A scientifically advanced approach involves fermentation-assisted isolation. This green chemistry technique utilizes lactic acid fermentation with specific starters (e.g., *Streptococcus thermophilus* or *Lactiplantibacillus plantarum*) to achieve the necessary pH reduction for isoelectric precipitation, replacing the use of harsh mineral acids [23]. This process significantly reduces anti-nutritional compounds, including trypsin inhibitor activity and phytic acid. The fermentation treatment demonstrably reduces bitterness while improving solubility and dispersibility. Sensory profile variations and presence of inherent pea-like bitterness have been linked to cultivar selection and isolation processes, underlining the need for optimized extraction protocols tailored to specific end uses.

**Figure 3:**
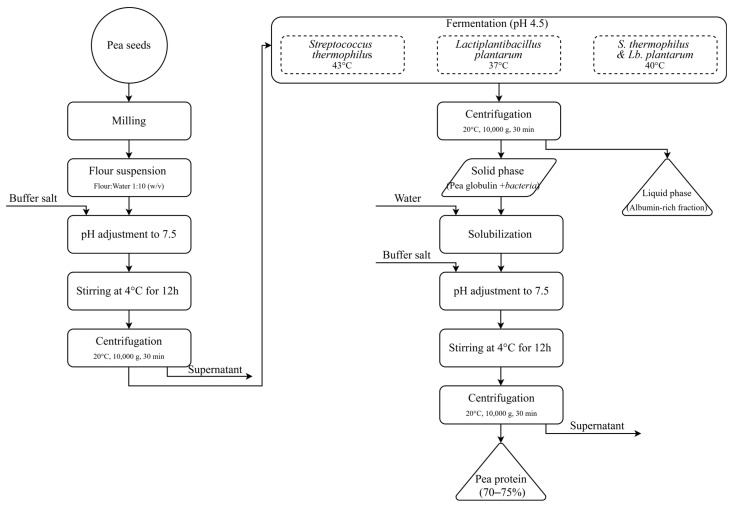
Schematic representation of fermentation-assisted extraction of pea protein, showing the combined effects of buffer solubilization and microbial processing.

Structurally, fermentation induces controlled degradation of proteins into small peptides and free amino acids, confirmed by size exclusion chromatography (SEC-HPLC). This results in a higher total nitrogen content in the soluble (albumin-rich) fraction, which consequently leads to elevated antioxidant activity in the resulting fraction [23]. This dual-purpose strategy establishes a robust link between controlled proteolysis during isolation and the generation of enhanced functional and bioactive benefits. The strategic opportunity exists to shift the focus of isolation toward controlled enzymatic or microbial hydrolysis, intentionally maximizing bioactive peptide production and moving the ingredient beyond bulk commodity status toward specialized nutraceutical and high-performance functional ingredients. To visualize this concept, Figure 3 presents the fermentation-assisted extraction pathway, combining solubilization and microbial processing to improve both recovery and biofunctional attributes of pea protein.

### 2.4. Impact of Isolation on Functionality

The extraction of soluble protein fractions from air-classified pea concentrates and their direct application in high-moisture extrusion have been shown to enhance gel elasticity and reduce brittleness of extruded products, indicating the importance of protein composition and solubility in texturization [30]. Pea protein extraction method impacts the protein (micro)structural organization and in vitro digestion kinetics, with processing-induced protein denaturation and aggregation detrimentally affecting solubility, emulsification, and gelation [27,31]. Variability in functional properties due to cultivar differences, processing parameters, and isolation techniques creates batch-to-batch inconsistencies, complicating standardization and scalability in industrial production. Consistency in protein purity and techno-functional attributes requires stringent control over isolation processes.

Scaling laboratory extraction methods to industrial levels while maintaining protein integrity and functionality remains a challenge. Control over the degree of denaturation, aggregation, and molecular structure during scale-up has a significant impact on the usability of resultant pea protein isolates in diverse applications. The isolation method selection represents the first critical decision in pea protein ingredient development, as it fundamentally determines the balance between purity and functionality, structural integrity versus yield considerations, and impacts all downstream functionality. There is no one-size-fits-all extraction method; rather, the choice must be guided by the intended application requirements and desired functional profile.

## 3. Structural Characteristics and Molecular Organization

The functionality of PPI is fundamentally dictated by the structure and ratio of its storage proteins, primarily globulins and albumins. Understanding these structural characteristics is essential for rational protein engineering and functional optimization.

### 3.1. Composition and Molecular Profile

**Figure 4:**
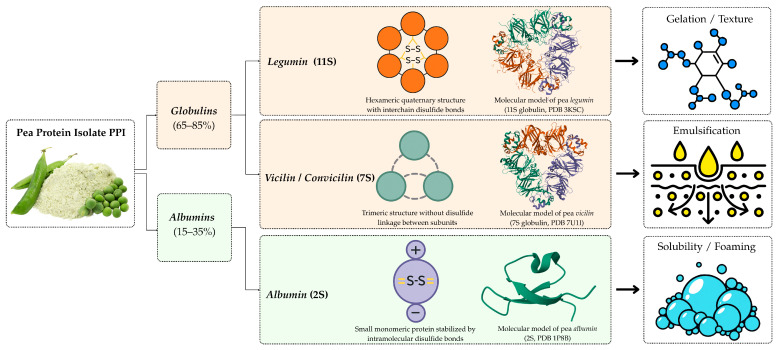
Hierarchical structure of pea proteins and associated functional properties.

Pea protein predominantly comprises three main globulin fractions: vicilin (7S), legumin (11S), and convicilin, accompanied by albumins [32]. Globulins constitute the majority of pea protein (65–85%), predominantly consisting of the high-molecular-weight 11S legumin and the lower-molecular-weight 7S vicilin and convicilin [28]. Vicilin and legumin constitute approximately 80–90% of the total pea seed protein, with varying ratios among cultivars affecting functional behavior. These globulins are trimeric and hexameric proteins, respectively, whose quaternary structures influence emulsifying and gelation properties. The structural organization of these protein fractions, from the molecular to the supramolecular level, plays a decisive role in defining their techno-functional properties. Figure 4 illustrates the hierarchical structure of pea proteins, linking their molecular architecture with corresponding functional behaviors such as solubility, emulsification, and gelation capacity.

Legumin (11S) is characterized as a hexameric protein, stabilized by internal disulfide bonds [33]. Vicilin and convicilin (7S) are generally trimeric. Albumins represent a smaller fraction and have distinct functional attributes associated with their smaller molecular size and higher surface charge [32]. Electrophoretic and size-exclusion chromatographic analyses reveal the heterogeneous composition concerning molecular weights, with major bands at around 50 kDa (vicilin), 60 kDa (legumin acidic subunit), and 20–30 kDa for albumins [23]. Protein profiles can be substantially affected by genotype and extraction conditions, impacting the concentration and ratio of these fractions.

The ratio between vicilin and legumin components is a key determinant of functional capacity, showing a positive correlation with protein extractability and beneficial functional properties, notably emulsification [32,34]. This ratio correlates with functional properties such as emulsifying activity and solubility. The performance of pea protein in interfacial applications is governed by its amphiphilicity—the balance between hydrophobic and hydrophilic regions. Certain isoforms of vicilins are naturally favored for interfacial activity because they possess superior amphiphilic balance [35].

### 3.2. Secondary and Tertiary Structure

Spectroscopic analyses (Fourier transform infrared spectroscopy, circular dichroism) provide insights into pea protein secondary structures characterized by varying proportions of α-helices, β-sheets, and disordered regions [36]. Structural modifications induced by processing or functionalization approaches alter these conformations, which are fundamental to the protein’s functional properties. Complexation with polyphenols such as epigallocatechin-3-gallate (EGCG) can lead to shifts from α-helix to β-sheet and increased disorder, resulting in modified thermal and emulsifying properties [36].

Upon emulsification, pea protein undergoes conformational changes, particularly in regions rich in tryptophan residues, though some commercial isolates exist as soluble aggregates with minimal modifications during adsorption to the oil–water interface [37]. Such structural behavior influences how the protein stabilizes emulsions, with unfolding exposing hydrophobic regions critical for interfacial activity. Controlled unfolding through physical or chemical treatments can facilitate enhanced functional characteristics. Protein unfolding and aggregation phenomena are documented, with treatments such as denaturants and modifiers inducing exposure of free amino groups, disulfide bonds, and changes in sulfhydryl content [38]. These molecular alterations significantly impact water and oil holding capacities, foaming, and emulsification, underscoring the intricate relationship between structure and function.

### 3.3. Denaturation and Aggregation Pathways

Pea proteins exhibit specific thermal aggregation pathways. Low-denatured legumin (approximately 350–410 kDa) and vicilin/convicilin (approximately 170 kDa) subunits undergo heat-induced denaturation, resulting in their reassociation into high-molecular-weight, soluble aggregates, which can exceed 700 kDa [33]. Thermal and enzymatic processing have a significant impact on the molecular structure of pea proteins. Heat treatment between 80 °C and 100 °C induces aggregation and denaturation, as evidenced by the formation of high-molecular-weight complexes (~200 kDa), resulting in altered emulsification capacity that depends on the pH and processing history [39].

A critical structural aspect is the role of disulfide bonds, which provide stability within the legumin subunits. Studies utilizing reducing agents, such as dithiothreitol, have shown that disrupting these linkages results in the formation of partially insoluble macroaggregates [33]. This observation highlights the crucial role of covalent bonds in maintaining the soluble nature of protein structure, whether in its native or aggregated form. Isolation processes, particularly harsh AE-IEP, must therefore be carefully controlled to minimize disruption of these key covalent linkages, as loss of native structure memory renders the resulting isolate less capable of forming desirable, stable macro-structures.

Heat-induced gelation occurs with the formation of networks stabilized by hydrophobic interactions and hydrogen bonding, which correlate with increased gel hardness and elasticity [40]. Water binding capacity correlates positively with gel texture, essential for maintaining product juiciness and mouthfeel. The gelation is heavily influenced by protein concentration and environmental pH, with variations in processing affecting the final gel strength and microstructure.

### 3.4. Structure–Function Relationships

The concentration of the protein suspension significantly influences the composition of the proteins adsorbed at the interface [35]. At low concentrations, the adsorption profile is ranked as aggregates > vicilin > legumin > convicillin. However, at saturated adsorption, the profile shifts, favoring vicilin > legumin > aggregates > convicillin. This suggests that at saturation, more flexible and amphiphilic molecular components (vicilin and legumin) can rapidly displace or mask larger, less mobile aggregates that initially dominate the adsorption layer.

Structural engineering efforts for PPI should be directed at optimizing the vicilin–legumin ratio or employing targeted modification to maximize the surface-exposed amphiphilic character essential for superior interfacial performance. The balance between adsorbed unfolded proteins and insoluble residue content significantly influences emulsion stability against droplet coalescence and flocculation [37,41]. Understanding these structure–function relationships provides the foundation for rational modification strategies to overcome native limitations and enhance desired functional properties.

## 4. Modification Strategies to Enhance Functional Properties

Structural engineering is necessary to overcome the native functional limitations of PPI. Modification techniques include enzymatic, physical, and chemical treatments designed to alter surface charge, hydrophobicity, and molecular conformation to improve functionality.

Table 2 summarizes the main enzymatic, physical, and chemical modification strategies applied to pea protein isolates, outlining their mechanisms, resulting structural alterations, and functional consequences. These techniques often act synergistically, with combined or sequential treatments providing greater control over solubility, gelation, and interfacial behavior than single interventions.

### 4.1. Enzymatic Modifications

#### 4.1.1. Enzymatic Hydrolysis and Protein Fragmentation

Enzymatic hydrolysis using proteases (papain, trypsin, chymosin) effectively improves solubility, emulsifying, and foaming characteristics by selectively cleaving peptide bonds [42]. However, the degree of hydrolysis must be carefully controlled, as excessive hydrolysis can lead to loss of gelling capacity and the generation of bitter peptides from hydrophobic amino acid residues. The challenge lies in identifying optimal enzyme-to-substrate ratios and reaction times that maximize functional improvements while preserving desirable textural properties and minimizing off-flavors. Chymosin-limited hydrolysis has shown promise in balancing these competing requirements, though cultivar-specific protein compositions necessitate tailored optimization protocols [54].

#### 4.1.2. Enzymatic Deamidation

Enzymatic deamidation using Protein Glutaminase converts neutral amide groups into negatively charged carboxyl groups, increasing electrostatic repulsion and enhancing dispersion stability [6,43]. A critical limitation is the enzyme’s cost and activity requirements, which can be pH- and temperature-sensitive, complicating industrial-scale implementation. Additionally, excessive deamidation may induce protein aggregation under certain processing conditions, particularly at intermediate degrees of modification where charge distribution becomes heterogeneous. The technique requires precise monitoring via zeta potential and FTIR analysis to ensure functional improvements are achieved without compromising protein structural integrity.

#### 4.1.3. Transglutaminase Cross-Linking

Transglutaminase (TGase) catalyzes covalent cross-links between glutamine and lysine residues, significantly improving gel strength and viscoelastic properties [44]. The primary challenge is substrate specificity—TGase preferentially targets disordered protein regions, and the compact globular structure of native pea proteins limits accessible reaction sites. Pre-treatments such as heat denaturation or pH-shifting are often required to expose reactive residues, adding process complexity. Furthermore, TGase activity is calcium-dependent and sensitive to inhibitors naturally present in plant matrices, requiring careful formulation control. The high cost of microbial TGase and consumer concerns about enzyme-modified ingredients also pose adoption barriers in clean-label applications.

### 4.2. Physical Modifications

#### 4.2.1. Ultrasound Treatment

High-intensity ultrasound induces cavitation effects that disrupt protein aggregates and promote controlled unfolding, enhancing emulsifying activity and interfacial properties [45]. However, process optimization is challenging due to multiple interdependent parameters, including frequency, power density, treatment duration, and temperature control. Excessive ultrasound exposure can cause irreversible protein denaturation and aggregation, reducing solubility rather than improving it. Energy consumption is substantial, and scale-up from laboratory to industrial systems introduces difficulties in maintaining uniform cavitation intensity throughout large volumes. Additionally, ultrasound-induced free radical formation may trigger protein oxidation, necessitating careful control of treatment conditions to avoid detrimental side reactions.

Synergistic combinations of ultrasound with pH-shifting have demonstrated enhanced solubility and gel strength [49]. While these combined approaches can overcome individual limitations, they also increase process complexity and require precise control of sequential treatment parameters to achieve reproducible results across different protein batches.

#### 4.2.2. High-Pressure Homogenization

High-pressure homogenization (HPH) improves solubility and emulsifying activity by inducing protein unfolding and modulating globulin subunit distribution [47,48]. A significant limitation is the high capital cost and energy requirements of industrial-scale HPH equipment operating at 100–150 MPa. Multiple passes through the homogenizer are often necessary to achieve optimal particle size reduction, further increasing energy consumption and processing time. The technique can also generate excessive heat, requiring efficient cooling systems to prevent thermal denaturation. Furthermore, the impact on gel strength is indirect—mediated through nanoemulsion droplet size—making it less effective for applications requiring strong protein gels formed directly from protein networks rather than emulsion-filled gels.

#### 4.2.3. Cold Plasma Treatment

Cold plasma treatment induces gel formation at reduced temperatures (70–80 °C) while improving gel mechanical strength and water-holding capacity [46]. Despite these advantages, the technology remains at a relatively early stage of industrial adoption. Key challenges include a lack of standardized equipment and protocols, making inter-laboratory comparison difficult. The reactive species generated by plasma can cause protein oxidation and lipid peroxidation if treatment parameters are not carefully controlled, potentially introducing off-flavors and reducing nutritional quality. Scale-up difficulties arise from maintaining uniform plasma exposure across large sample volumes, and the batch-wise nature of most plasma systems limits throughput compared to continuous processing technologies. Consumer acceptance may also be limited by unfamiliarity with plasma technology in food applications.

### 4.3. Chemical Modifications

#### 4.3.1. Succinylation

Succinylation introduces negative charges to lysine residues, enhancing solubility and emulsifying stability [50,51]. However, chemical modification techniques face significant regulatory and consumer acceptance challenges, particularly in markets demanding clean-label products. Succinylation requires the use of succinic anhydride, raising concerns about chemical residues and the perception of heavily processed ingredients. The reaction must be carefully controlled to avoid over-modification, which can compromise protein nutritional value by blocking essential lysine residues. Removal of excess reagents and by-products adds downstream processing complexity and cost. Additionally, succinylated proteins may exhibit altered digestibility and bioavailability compared to native proteins, requiring thorough nutritional characterization.

#### 4.3.2. Glycation and Polyphenol Complexation

Glycation via Maillard-type reactions enhances emulsifying performance and thermal stability [43,52]. The principal challenge is controlling reaction conditions to achieve glycation without excessive browning or formation of advanced glycation end-products (AGEs), some of which have been associated with adverse health effects. The reaction kinetics are highly dependent on temperature, time, pH, and sugar-to-protein ratio, making reproducible production difficult without precise process control. Ultrasound-assisted glycation can accelerate reactions but introduces additional complexity in optimizing combined process parameters. Furthermore, glycation blocks lysine residues, potentially reducing protein nutritional quality and requiring careful balancing of functional improvements against nutritional losses.

Polyphenol complexation with compounds like EGCG improves foaming and emulsifying properties while adding antioxidant functionality [36]. Challenges include the high cost and limited availability of purified polyphenols, potential interactions with other food components that may destabilize complexes, and the impact of polyphenols on color and flavor profiles. The noncovalent nature of these complexes makes them susceptible to dissociation under processing conditions or in complex food matrices, limiting their application range.

### 4.4. Combined and Synergistic Approaches

The most effective modification strategies often involve combinations of multiple treatments to achieve synergistic effects [53]. For example, combining enzymatic hydrolysis with physical treatments can provide complementary structural changes that address multiple functional limitations simultaneously. However, multi-step modification sequences substantially increase process complexity, capital investment, and operating costs. Each additional processing step introduces potential sources of variability and requires optimization of treatment order and intermediate handling conditions. The cumulative impact on protein nutritional quality must be carefully evaluated, as sequential modifications may have additive effects on essential amino acid availability. Furthermore, regulatory approval pathways become more complex for multiply-modified ingredients, and consumer acceptance may be lower for products perceived as heavily processed. Despite these challenges, targeted combination strategies guided by structure–function principles offer the most promising route to achieving functional parity with animal proteins while maintaining acceptable costs and clean-label positioning.

## 5. Functional Properties: Gelation, Emulsification, and Stabilization

### 5.1. Gelation Properties and Mechanisms

#### 5.1.1. Intrinsic Gelation Limitations

Unmodified pea protein isolates typically yield weak gels compared to animal proteins [25]. Scientific studies defining the conditions necessary to achieve strong hydrogels—defined by an elastic modulus (G’) greater than 10^3^ Pa and the ability to retain over 90% water—show that PPI requires highly specific, intensive formulation parameters [25]. These critical conditions include high concentration (at least 20% *w*/*w*), a pH substantially removed from the isoelectric point, and a high ionic strength (IS above 0.6 M). The globular, compact structure of native PPI inherently limits efficient chain entanglement and intermolecular interaction necessary for forming a dense, load-bearing network.

Rheological analysis confirms the inherent mechanical weakness. The maximum strain resistance within the linear viscoelastic regime (LVR) for PPI is extremely low, ranging from 0.74% to 1.26% [54,55]. This poor strain resistance falls significantly below the threshold required for strong, robust gels (γ = 25%), indicating a rapid loss of structure and a tendency toward flow behavior. This deficiency confirms that the globular, compact structure of native PPI inherently limits efficient chain entanglement and intermolecular interaction necessary for forming a dense, load-bearing network.

#### 5.1.2. Enhancement Strategies

The addition of polysaccharides such as carrageenan strengthens gel matrices by forming coupled networks with pea proteins, enhancing elasticity up to 75-fold and increasing water retention [44]. This cross-linked microstructure modulates textural and digestive behavior, demonstrating the potential for precise design of gel-based foods with targeted functional and nutritional profiles. The identification of the specific environmental requirements (concentration, pH, ionic strength) necessary for gelation allows for the construction of a predictive gelation map [25]. This map is crucial for modulating the rheological properties of pea protein hydrogels, which serve as templates for subsequent advanced materials science processes, including their conversion into xerogels, cryogels, and aerogels.

### 5.2. Emulsification Characteristics

#### 5.2.1. Intrinsic Emulsifying Properties

The effectiveness of PPI in liquid and multiphase systems, particularly emulsions, is dictated by its ability to rapidly and efficiently adsorb at the oil–water interface and subsequently stabilize the resulting droplets against coalescence. Pea protein’s intrinsic emulsifying properties stem from its amphiphilic nature, surface hydrophobicity, solubility, and molecular size [6]. Functional solubility is crucial; greater solubility typically correlates with enhanced water-holding and emulsifying activity indices (EAI). Pea protein’s emulsification potential covers a range of pH conditions, with solubility and emulsion droplet size being modulated by pH, often presenting optimal function near neutral pH. Effective interfacial activity requires a favorable balance of hydrophobic and hydrophilic groups (amphiphilicity) in the protein sequence. The ratio of vicilin to legumin concentrations is positively associated with high extractability and superior emulsion activity index (EAI) [32,34]. Furthermore, EAI is strongly and positively correlated with the protein’s overall solubility [32]. Emulsion droplet size and stability are sensitive to processing conditions such as drying, temperature, and exposure to hydrophobic interaction inhibitors.

Comparative analyses reveal differences between pea proteins and traditional dairy proteins like whey, with plant proteins generally forming larger droplets and less stable emulsions under certain conditions [9,37]. Nonetheless, under appropriate modifications, pea protein emulsions can match or even surpass some plant and animal proteins in emulsifying efficiency, facilitated by molecular unfolding and increased surface hydrophobicity.

#### 5.2.2. Mechanisms of Emulsion Stabilization

Pea proteins stabilize emulsions through multiple mechanisms, including Pickering stabilization by protein particles, as well as classical adsorption and unfolding at the oil–water interface. Studies on self-assembled pea protein particles demonstrate partial coverage of oil droplets, with a significant contribution coming from molecularly dissolved protein species adsorbing at interfaces [56]. Thus, both particulate and molecular forms operate synergistically to provide stabilization. Protein unfolding upon emulsification exposes hydrophobic residues, enabling tight interfacial adsorption. However, some commercial isolates consist mainly of soluble aggregates that do not show tryptophan conformational changes upon adsorption, suggesting diverse structural behaviors depending on isolate preparation [37,41].

A critical mechanistic distinction has been established regarding droplet stabilization, particularly at acidic pH. Studies conducted at pH 3.0 demonstrate that the stabilization mechanism in pea proteins is based primarily on protein molecules rather than particles [56]. Evidence indicates that protein particles or large aggregates do not play a major role in droplet surface stabilization at this pH. This observation has profound implications for formulation strategy, confirming that for applications requiring stability at low pH, such as acidic beverages, success depends on preserving or maximizing soluble molecular components rather than engineering hard particle stabilizers. Emulsions stabilized by pea proteins maintain consistent properties across a wide pH range from acidic to neutral, highlighting the robustness of pea protein interfacial films under variable food processing and storage conditions [31,39].

#### 5.2.3. Effects of Processing on Emulsifying Capacity

Protein oxidation adversely affects emulsion stability by increasing fragmentation and generating low-molecular-weight products that disrupt droplet integrity and promote coalescence [57]. Microfluidic studies reveal that mildly oxidized proteins form heterogeneous interfacial films, which are structurally weak, whereas freshly prepared or less oxidized proteins yield more homogeneous, stable emulsions. Physical pretreatments such as ultrasound-assisted glycosylation with polysaccharides enhance emulsifying activity by increasing molecular flexibility and surface hydrophobicity, improving protein adsorption and interfacial film formation.

Pretreatment of pea protein isolates influences protein unfolding and aggregation, which affects emulsion droplet size and stability. Heating above denaturation temperature facilitates emulsion formation but may reduce solubility and foamability, stressing the importance of controlled thermal regimes [29,39]. However, denaturation induced by thermal treatment can adversely affect stability, highlighting the importance of controlled processing temperatures to preserve protein functionality.

### 5.3. Foaming Properties

Foaming properties represent a critical techno-functional characteristic of pea protein isolates, which defines their application in aerated food products such as mousses, meringues, whipped toppings, and bakery goods. Foam formation and stability depend on the protein’s ability to rapidly adsorb at the air–water interface, unfold to expose hydrophobic residues, and form cohesive interfacial films that resist bubble coalescence and disproportionation. Native pea protein isolates exhibit moderate foaming capacity compared to conventional foaming agents like egg white or whey protein [32]. The globular structure of legumin and vicilin fractions limits their interfacial flexibility and rate of adsorption at air–water interfaces. Studies on different pea genotypes reveal that while foaming capacity shows limited variability across cultivars, foaming stability is more sensitive to protein composition, particularly the albumin-to-globulin ratio [32]. Albumin fractions demonstrate superior foaming characteristics, achieving foam overrun values up to 258% with extended stability (>270 min), attributed to their smaller molecular size and ability to form robust, cohesive interfacial layers at the air–water interface [23]. In contrast, globulin-rich fractions yield considerably lower foam overrun (<81%) and reduced stability (<70 min) due to weaker, more mobile interfacial films formed by larger, highly charged protein aggregates.

Thermal pretreatment significantly impacts foaming functionality. Heat treatment above the denaturation temperature (80–100 °C) induces protein aggregation and formation of high-molecular-weight complexes that are excessively large to stabilize foams effectively, resulting in a marked reduction in foaming properties regardless of pH conditions [39]. This limitation highlights a critical trade-off, as thermal processing often improves emulsification but compromises foaming capacity.

Modification strategies can enhance foaming performance. Enzymatic hydrolysis using proteases such as chymosin improves foam stability by reducing molecular weight and increasing interfacial film flexibility [54]. Polyphenol complexation with compounds like EGCG alters secondary structure and enhances foaming activity while providing antioxidant functionality [36]. However, optimization remains challenging, as treatments that improve solubility and emulsification may not proportionally enhance foaming, necessitating application-specific modification protocols tailored to the desired foam characteristics and stability requirements.

### 5.4. Stabilization Factors and Interfacial Behavior

#### 5.4.1. Emulsion Stability Index and Long-Term Performance

**Figure 5:**
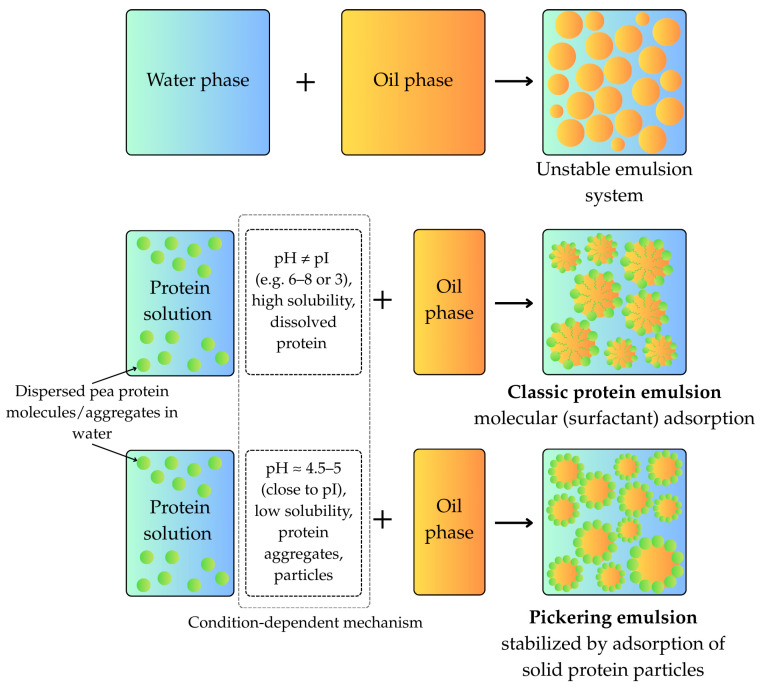
Mechanistic pathways of pea protein emulsification and stabilization.

The Emulsion Stability Index (ESI) quantifies the protein’s ability to prevent the separation of the resulting emulsion over time. While EAI correlates strongly with solubility, scientific investigations have found no significant correlation between ESI and solubility [32]. This distinction indicates that initial emulsification performance (EAI) and long-term stability (ESI) are governed by different structural and interfacial properties. Long-term stability depends more on the strength and reorganization capacity of the interfacial film rather than simple solubility. Figure 5 summarizes the mechanistic pathways of pea protein emulsification and stabilization, showing key interfacial and molecular processes responsible for emulsion formation and long-term stability.

#### 5.4.2. Environmental and Compositional Influences

The capability to produce stable, high-internal-phase emulsions through processing modulation expands the use of pea protein in sauces, dressings, and functional beverages [48,56]. Interfacial properties and antioxidant capacity of Pickering emulsions can be enhanced through the formation of complexes with high methoxyl pectin and curcumin, demonstrating multifunctional ingredient design possibilities [58]. Physical and emulsifying properties can be further improved through combined physical modification by flaxseed gum and ultrasonic treatment [59].

## 6. Advanced Food Applications and Emerging Technologies

### 6.1. Meat Analogues and Texturized Products

Pea proteins have been extensively applied in the development of plant-based meat analogues, where their gelation and water absorption behaviors are utilized during extrusion to create texturized structures mimicking meat fiber [53]. Variations in the physicochemical properties of pea protein isolates, such as cold swelling ability, gelation concentration, and thermally induced phase transition, guide the formulation for optimal extrusion performance and final product texture. Enhancement of gel elasticity through soluble protein fractions improves the mechanical properties of extrudates, reducing brittleness and contributing to a more meat-like texture [30].

Cold-set gels produced with pea protein-based binders exhibit controllable gel strength and water-holding capacity, which are critical for product cohesion and sensory experience in meat alternatives [30,60]. pH-shifting processes improve the gelling properties of pea protein and demonstrate potential application as binders in meat alternative products. Understanding functional properties such as water absorption, least gelation concentration, and phase transition temperature has facilitated the production of texturized products with tailored textural properties, with differences in protein cross-linking potential and cold swelling behavior dictating the energy input required during extrusion and the final product structure [53].

### 6.2. Emulsion-Based Products and Delivery Systems

Pea protein stabilizes emulsions ranging from macro- to nanoemulsions and supports the formation of Pickering emulsions, providing stable delivery systems for food and nutraceutical applications [48,56]. The capability to produce stable high internal phase emulsions through processing modulation expands their use in sauces, dressings, and functional beverages. Pea protein matrices have been investigated for microencapsulation and controlled release of flavors and bioactives. Advances in microencapsulation technology incorporate pea protein as a sustainable wall material with functional emulsifying and stabilizing properties, enabling tailored release profiles in foods [61].

The formation of complexes with polysaccharides and bioactive compounds enhances both functionality and nutritional value. Interfacial properties and antioxidant capacity of Pickering emulsions stabilized by high methoxyl pectin–surfactant–pea protein isolate–curcumin complexes demonstrate the potential for multifunctional ingredient design [58]. These systems provide not only structural functionality but also deliver health-promoting compounds in a stable and bioavailable form.

### 6.3. Edible Films and Biodegradable Materials

The formation of pea protein-based edible films and composites with polysaccharides such as sodium carboxymethyl cellulose offers promising packaging materials with improved mechanical strength, water resistance, and thermal stability [62]. These films have been effectively applied for food preservation, reducing moisture transfer, and improving shelf life. Quality improvement of pea protein isolate-based films through incorporation of sodium carboxymethyl cellulose has demonstrated enhanced barrier and mechanical properties, with specific formulations showing marked improvements in water vapor permeability, water contact angle, tensile strength, and elongation at break [62].

The effects of high-intensity ultrasound on the structural, optical, mechanical, and physicochemical properties of pea protein isolate-based edible films have been investigated, demonstrating that ultrasound treatment can modify film properties for specific applications [63]. Biodegradable active composite films based on pea protein isolate, sage seed gum, and cumin essential oil demonstrate fabrication and characterization approaches for multifunctional packaging materials [64]. Edible films manufactured from pea proteins have been evaluated for their mechanical strength and physicochemical features, with studies comparing performance against other protein sources [65].

### 6.4. Dairy Alternatives and Fermented Products

Pea protein demonstrates promising functionality in dairy alternative applications, including plant-based yogurts and beverages. The physicochemical properties of different pea proteins in relation to their ability to form a lactic acid bacteria-induced yoghurt gel have been characterized, showing potential for fermented product development [40]. The ability to form stable gels under acidic conditions makes pea protein suitable for yoghurt-type products, where gel strength and texture are critical quality parameters.

The impact of κ-carrageenan on cold-set pea protein isolated emulsion-filled gels has been studied in terms of mechanical property, microstructure, and in vitro digestive behavior, providing insights for dairy alternative product formulation [44]. These composite systems offer the opportunity to tailor texture, nutritional profile, and digestive characteristics to match or exceed those of traditional dairy products, while providing plant-based alternatives for consumers with dietary restrictions or preferences.

### 6.5. Nutraceutical and Bioactive Applications

Pea protein hydrolysates and peptides exhibit bioactive properties, including antioxidant and antihypertensive effects, as well as modulation of intestinal bacteria [2]. This health benefits position pea protein as more than a nutritional ingredient, extending its role into nutraceuticals and functional foods designed for targeted health outcomes. The fermentation-assisted extraction approach, which yields albumin-rich fractions with enhanced antioxidant activity, represents one pathway to producing high-value bioactive ingredients [23].

The integration of pea protein into functional food formulations enhances nutritional quality by providing high-quality plant protein and bioactive peptides, thereby contributing to improved consumer health and positioning the ingredient within health-driven food markets [7]. The strategic opportunity exists to move pea protein beyond bulk commodity status toward specialized nutraceutical ingredients through controlled processing that maximizes bioactive peptide generation. This approach supports the evolution of pea protein from a simple protein source to a platform for creating designer functional ingredients with specific health-promoting properties.

### 6.6. Challenges in Application Development

Despite advances, pea proteins often present challenges, such as relatively poor solubility and emulsification under acidic conditions and in certain food matrix environments. Off-flavor attributes, notably characteristic pea-like bitterness, can detract from consumer acceptance and limit broader utilization in sensory-sensitive food products [2,8]. Variability in functional properties due to cultivar differences, processing parameters, and isolation techniques creates batch-to-batch inconsistencies, complicating standardization and scalability in industrial production.

To address solubility and emulsification limitations under acidic conditions, targeted solutions include pH-shifting pretreatment combined with ultrasound (8–15 kHz, 20–30 min) to enhance surface hydrophobicity and interfacial adsorption [49], succinylation to introduce anionic groups that maintain charge repulsion at low pH [51], and microfluidization at 100–150 MPa to produce protein nanoparticles with improved acid stability [48]. For off-flavor mitigation, enzymatic debittering using endo- and exo-peptidases selectively hydrolyzes bitter peptides while preserving functional regions [54], fermentation with Lactobacillus strains metabolizes volatile aldehydes and ketones responsible for beany notes [23], and flavor masking through Maillard conjugation with glucose or xylose (60–80 °C, controlled humidity) creates flavor-neutral glycoproteins suitable for beverage applications [53].

Batch-to-batch variability can be minimized through the implementation of rapid protein profiling using size-exclusion chromatography to quantify legumin-to-vicilin ratios and adjust processing parameters accordingly [32], adoption of statistical process control with critical quality attributes including protein solubility at pH 7.0, emulsion activity index, and gel strength as standardized metrics [61], as well as the development of cultivar-specific extraction protocols that account for genotype-dependent compositional differences in globulin distribution and anti-nutritional factor content [34]. Market trends toward plant-based, sustainable, and clean-label products drive the demand for pea protein ingredients with optimized technical and sensory characteristics, supporting continued innovation and commercial growth [7].

## 7. Conclusions

This review establishes three core contributions to the understanding of pea protein functionality: (1) a structure–function framework linking globulin conformation to performance limitations in gels, emulsions, and films; (2) a systematic classification of extraction technologies based on their impact on protein structural integrity and downstream functionality; and (3) an evidence-based categorization of modification strategies according to their mechanistic targets—surface charge manipulation, structural unfolding, or covalent cross-linking—with quantitative performance benchmarks for each approach.

The primary scientific challenge lies in the intrinsic weakness of PPI macro-structures, such as gels and films, under standard processing conditions. This weakness is a direct consequence of the compact, globular conformation of the storage proteins (legumin and vicilin), which limits their capacity for efficient intermolecular entanglement and non-covalent network formation [25]. Functional performance metrics—including Emulsion Activity Index, elastic modulus (G’), and film tensile strength—are critically dependent on moving away from isolation protocols solely focused on maximum intact protein yield toward strategies centered on targeted structural control.

Mechanistic understanding of protein unfolding, aggregation, and interfacial adsorption provides insights into tailoring pea protein functionality for specific food applications. Modification strategies, including enzymatic hydrolysis, ultrasound, glycation, pH-shifting, and transglutaminase cross-linking, have proven effective in overcoming innate limitations [38,46]. Methodologies that enhance solubility and molecular amphiphilicity (e.g., succinylation or ultrasound treatment) or enforce robust networks (e.g., Transglutaminase cross-linking) are essential for successful industrial adoption in applications that demand high functional reliability.

The necessity for extensive engineering arises from the limitations of the native structure. The native pea protein structure dictates both its limitations and the engineering strategies required to overcome them. Isolation as the first critical decision establishes the purity–functionality trade-off, with method selection impacting all downstream functionality. Each functional limitation has corresponding engineering solutions through enzymatic, chemical, and physical tools, with synergistic combination strategies offering enhanced results through structure-guided design principles.

For instance, achieving strong gelation requires forcing the system to high concentration (20%), high pH, and high ionic strength [25]. Similarly, biomaterial applications require cross-linking to enforce a dense, load-bearing structure to achieve the mechanical performance of natural polymers like gelatin [65]. The identification of specific environmental requirements necessary for gelation allows for the construction of predictive gelation maps critical for modulating rheological properties of pea protein hydrogels used as templates for advanced materials science processes.

Pea protein is emerging as a promising alternative to animal proteins in diverse food products due to functional and nutritional attributes combined with sustainability advantages. Nutritional parity or superiority is achievable in terms of PDCAAS and amino acid profile, with functional gaps in gelation and emulsification having been narrowed through engineering approaches [6]. Remaining challenges include matching sensory properties and texture in specific applications, as well as balancing cost and performance trade-offs for modified ingredients.

Scalability and economic considerations involve the maturity of industrial-scale extraction technology, the cost of modification techniques, clean-label and consumer acceptance factors, and navigating the regulatory landscape [27,31]. Sustainability advantages include water and nitrogen use efficiency, reduced carbon footprint versus animal proteins, crop rotation benefits, and circular economy potential through valorization of all protein fractions [7].

Future research must prioritize integrated biorefinery concepts. The current industry focus on the globulin-rich isolate often undervalues the soluble albumin fraction. However, this fraction, when extracted via green techniques like lactic acid fermentation, is significantly enriched with valuable components, including ANF-reduced, antioxidant peptides [23]. A strategic approach involves optimizing globulin purity for bulk food ingredients while directing the soluble peptide stream toward high-value nutraceutical and functional markets through dual-stream valorization models.

Future work should emphasize the development of mild, green extraction methods that better preserve protein native structures and functionality [8,27]. Integration of multi-scale structural characterization with functional studies will elucidate structure–function relationships that underpin product performance. Novel enzyme discovery and engineering, precision fermentation approaches, emerging physical technologies (cold plasma, pulsed electric field), and green chemistry innovations offer pathways for advanced modification technologies.

Sensory and consumer acceptance remain critical areas requiring off-flavor reduction strategies, beany/green note masking approaches, texture optimization for meat and dairy analogues, and comprehensive consumer perception studies. Standardization and characterization needs include development of standardized assessment protocols, improved reproducibility across studies, benchmarking frameworks, and quality control metrics.

## Figures and Tables

**Table 1:** Comparison of compositional, functional, and sustainability attributes of pea protein and selected reference proteins.

Protein Sample	[g/100 g Protein] Lysine; Leucine PDCAAS; DIAAS	Allergenicity/ Hypoallergenicity	Technological Limitations/ Functional Properties	Environmental Sustainability	Refs.
Pea Protein (Isolate, PPI)	~4.7–5.7; ~5.7–6.4; ~0.81–0.93; ~0.73–0.82	Low allergenicity. Pea proteins (legumin and vicilin fractions) are generally considered non-allergenic and suitable for hypoallergenic formulations, with only limited cross-reactivity reported with other legumes.	Limited solubility and weak gelation capacity due to globular structure; requires enzymatic or physical modification to improve emulsification and gelling.	Environmentally favorable. Low water and nitrogen fertilizer demand; positive rotational crop effects; typically, non-GMO.	[6,10]
Soy Protein (Isolate, SPI)	~6.3–6.5; 8.0; ~0.98–1.00; ~0.90–1.10	Recognized food allergen. Soy allergy affects ~0.4–0.6% of the population; it is more common in children and individuals who are already sensitized.	Strong functional performance in emulsification and foaming is primarily driven by glycinin (11S) and β-conglycinin (7S); functionality is process- and pH-dependent.	Variable footprint. High yields are achieved, but land-use impacts vary by region; both GM and non-GM cultivars are common; sustainability depends on the sourcing of inputs.	[11,12,13,14,15]
Wheat Protein (Gluten/WPI)	~2.6–2.8; 6.8; ~0.40–0.54; ~0.40–0.56	High allergenicity. Contains gliadin and glutenin, responsible for celiac disease, wheat allergy, and non-celiac gluten sensitivity.	Excellent viscoelastic network formation (gluten structure) is achieved; however, solubility is low and emulsifying ability is poor, making it suitable mainly for bakery applications.	Moderate footprint. High yield per hectare; mostly non-GMO; fertilizer demand can be significant.	[10,11,16,17]
Rice Protein (Isolate/ Concentrate)	~1.4–3.3; ~4.62–6.53; ~0.42–0.64; ~0.37–0.60	Very low allergenicity. Rice protein is well-tolerated and used in infants and hypoallergenic formulas; allergic reactions are rare	Poor solubility and weak emulsification, but good film-forming and digestibility properties; functionality can be improved via enzymatic hydrolysis or pH adjustment.	Moderate to high footprint. Water-intensive cultivation but lower GHG emissions than animal proteins.	[10,13,18,19,20]
Whey Protein (Isolate/ Concentrate)	~8.8–10.3; ~10.7–13.0; 1.0 (WPI/WPC); 1.09 (WPI) 0.97 (WPC)	High allergenicity. Common in individuals with cow’s milk protein allergy (CMPA); β-lactoglobulin and α-lactalbumin are major allergens.	Excellent solubility, emulsification, foaming, and gelation; functional benchmark for food proteins, but heat-sensitive above ~70 °C.	High environmental footprint. Derived from dairy; 5–10× higher GHG emissions and land use than plant proteins.	[13,21,22]

PDCAAS and DIAAS are dimensionless indices of protein quality.

**Table 2:** Overview of enzymatic, physical, and chemical modification techniques applied to pea protein isolates (PPI), their underlying mechanisms, structural effects, and impacts on key techno-functional properties.

Modification Technique		Mechanism	Structural Changes	Functional Impact	References
Enzymatic modifications	Enzymatic hydrolysis (papain, trypsin, chymosin)	Proteolytic cleavage of peptide bonds; controlled (limited) hydrolysis to tailor molecular weight distribution	Reduced molecular weight; broadened peptide-size distribution; disruption of allergenic epitopes; partial unfolding	↑ Solubility, ↑ emulsifying & foaming capacity, ↓ allergenicity, ↓ bitterness	[42]
	Enzymatic deamidation (Protein Glutaminase)	Conversion of amide groups (Gln, Asn) → carboxyl (Glu, Asp)	Increased negative charge, higher zeta potential, altered FTIR amide I and II bands	↑ Dispersion stability, ↑ solubility, ↑ heat resistance (due to reduced aggregation at elevated temperatures)	[6,43]
	Transglutaminase cross-linking (TGase)	TGase forms ε-(γ-glutamyl)lysine covalent cross-links between peptides/proteins	Covalent network formation; denser gel matrix; increased viscoelastic moduli (G′, G″)	↑ Gel strength, ↑ elasticity, ↑ structural stability	[44]
Physical modifications	Ultrasound (high-intensity)	Acoustic cavitation disrupts noncovalent interactions and aggregates	Partial unfolding of tertiary structure; disruption of weak aggregates; increased exposure of hydrophobic and sulfhydryl (–SH) groups enhances interfacial adsorption	↑ Emulsifying activity, ↑ flexibility, ↑ emulsifying & foaming activity, ↑ interfacial adsorption	[45]
	Ultrasound + pH-shifting (synergistic)	Extreme pH induces unfolding/charge redistribution; ultrasound reduces particle size and stabilizes the unfolded state	Pronounced unfolding of globulins; reduced particle size; increased surface charge; partial rearrangement of secondary-structure elements	↑ Solubility, ↑ gel strength, ↑ emulsion/interfacial stability due to enhanced protein–protein and protein–water interactions	[46]
	High-pressure homogenization (HPH)	High shear/pressure induces unfolding and subunit reorganization; disrupts aggregates	Reduced aggregate size; modulation of 7S/11S subunit abundance; partial tertiary-structure	↑ Solubility, ↑ oil-holding capacity, ↑ emulsifying activity; control of gel elasticity via droplet-size reduction in PPI-stabilized emulsions	[47,48]
	Cold plasma (non-thermal)	Reactive species induce mild unfolding and promote cross-link formation at lower temperatures	Partial tertiary unfolding; increased accessible –SH; formation of new covalent/noncovalent cross-links	↑ Gel strength, ↓ gelation temperature, ↑ water-holding capacity, ↑ elasticity	[49]
Chemical modifications	Succinylation	Covalent addition of succinyl groups to ε-NH_2_ of lysine residues	Increased net negative charge; lower isoelectric point; reduced protein–protein attractive interactions; more ordered α-helix/β-sheet content	↑ Solubility, ↑ emulsifying stability, smaller effective particle size in dispersion	[50,51]
	Glycation (Maillard-type; incl. ultrasound-assisted)	Covalent conjugation of reducing sugars to Lys/N-termini; ultrasound can accelerate reaction	Masked lysine residues; increased surface hydrophobicity; increased disorder; reduced particle size of conjugates; altered secondary structure	↑ Emulsifying and thermal stability; formation of stable Pickering HIPEs; ↑ oxidation stability	[43,52]
	Polyphenol complexation (e.g., EGCG)	Noncovalent complexes via H-bonding and π–π interactions	Altered secondary structure; increased hydrophilicity; minor conformational compaction/loosening depending on ligand ratio	↑ Foaming & emulsifying activity, ↑ antioxidant stability of emulsions	[36]
	Combined/synergistic modifications	Sequential or concurrent integration of enzymatic, physical, and/or chemical treatments	Multi-modal effects (charge redistribution, partial unfolding, cross-link modulation)	Tailored solubility, gelation, and texture; process efficiencies specific to combination	[53]

↑—increase, ↓—allergenicity.

## Data Availability

No new data were created or analyzed in this study. Data sharing is not applicable to this article.
